# Participant engagement to develop report-back materials for personal air monitoring

**DOI:** 10.1017/cts.2023.30

**Published:** 2023-03-03

**Authors:** Patrick H. Ryan, Chris Wolfe, Allison Parsons, Cole Brokamp, Ashley Turner, Erin Haynes

**Affiliations:** 1 Department of Pediatrics, University of Cincinnati, College of Medicine, Cincinnati, OH, USA; 2 Division of Biostatistics and Epidemiology, Cincinnati Children’s Hospital Medical Center, Cincinnati, OH, USA; 3 Division of Critical Care Medicine, Cincinnati Children’s Hospital Medical Center, Cincinnati, OH, USA; 4 Rescue Agency, San Diego, CA, USA; 5 Department of Epidemiology, University of Kentucky, College of Public Health, Lexington, KY, USA

**Keywords:** Participant engagement, reporting study findings to participants, personal air monitoring, focus group, communication

## Abstract

**Background:**

Studies that measure environmental exposures in biological samples frequently provide participants their results. In contrast, studies using personal air monitors do not typically provide participants their monitoring results. The objective of this study was to engage adolescents who completed personal air sampling and their caregivers to develop understandable and actionable report-back documents containing the results of their personal air sampling.

**Methods:**

Adolescents and their caregivers who previously completed personal air sampling participated in focus groups to guide the development of report-back materials. We conducted thematic analyses of focus group data to guide the design of the report-back document and convened experts in community engagement, reporting study results, and human subjects research to provide feedback. Final revisions to the report-back document were made based on follow-up focus group feedback.

**Results:**

Focus groups identified critical components of an air-monitoring report-back document to include an overview of the pollutant being measured, a comparison of individual personal sampling data to the overall study population, a guide to interpreting results, visualization of individual data, and additional information on pollution sources, health risks, and exposure reduction strategies. Participants also indicated their desire to receive study results in an electronic and interactive format. The final report-back document was electronic and included background information, participants’ results presented using interactive maps and figures, and additional material regarding pollution sources.

**Conclusion:**

Studies using personal air monitoring technology should provide research participants their results in an understandable and meaningful way to empower participants with increased knowledge to guide exposure reduction strategies.

## Introduction

Wearable sensors that measure personal exposure to air pollution represent a paradigm shift in exposure assessment and epidemiologic research. In contrast to stationary air monitoring, these devices provide users their individual-level information regarding exposure and can identify specific locations and activities associated with increased exposures, including being indoors, in transit (car, bus, and walking), and other settings [1,2]. Wearable air pollution monitors can also be linked to spatiotemporal geolocations and paired with other sensors to capture physiologic (e.g., heart rate variability and lung function) and physical activity (e.g., accelerometer) data, thereby connecting personal exposure data with individual health indicators [3]. As the reliability and accuracy of low-cost air monitoring devices continue to improve their use is expected to become widespread in research studies and by citizen-scientists who seek information regarding personal pollutant exposures [4].

While personal air monitors will provide new opportunities to researchers, the individual-level data they measure requires novel human subject considerations. In contrast to stationary air monitoring or statistical prediction models to estimate air pollution exposure, personal air monitors provide individual level and actionable information. Participants in studies that measure environmental exposures and chemicals in biospecimens report a desire to receive their results, and researchers agree that participants have a right to their individual results [5–8]. Effective report back of biomonitoring results also increases participants’ engagement with research, and guidelines for providing individual results to participants in biomonitoring studies are now available [9–11]. Biomonitoring studies have also demonstrated that engaging with communities and research participants in the development of report-back materials is critical to effectively develop report-back materials that are appropriate and specific to the study objectives and population [5,12]. There are critical differences, however, between biomonitoring studies and personal air monitoring and, to our knowledge, studies involving children and adolescents using personal air monitors have not examined participants’ desire to receive their exposure results nor considered best practices to return these results using an understandable and actionable approach.

Therefore, the objective of this study was to engage with participants and caregivers enrolled in the Ecological Momentary Assessment and Personal Particle Exposure (EcoMAPPE), a panel study of adolescents’ personal exposure to ultrafine particles (UFPs) and respiratory health, to determine appropriate methods to provide personal air monitoring results to research study participants. In addition, we applied qualitative research methods to identify the perceived benefits, concerns, and desired content and format for reporting personal air monitoring results back to participants and their caregivers.

## Materials and Methods

### Overview of Report-Back Development

We employed an iterative, participant-engaged approach to develop personal air monitoring report-back materials that included focus groups of adolescent study participants who completed a study of personal air sampling and their caregivers. In addition, we sought input from an expert panel of researchers experienced in community-engagement, reporting study results, and human subjects research regulatory affairs. Following the initial development of materials, we reconvened our focus groups prior to making final changes to the materials.

### Study Population

We enrolled a subset of adolescents (n = 8) who participated in the EcoMAPPE study and their caregivers (n = 8) to participate in separate focus groups for this study. Briefly, the EcoMAPPE study enrolled 118 adolescents aged 13–17 years to complete personal air monitoring and respiratory health assessments [13]. Personal exposure to UFPs (particulate matter < 100 nm in aerodynamic diameter) was measured during a one-week sampling campaign using the Personal Ultrafine Particle Counter (PUFP C200, EnMont LLC, Cincinnati, OH, USA), a wearable condensation particle counter with a built-in global positioning system (GPS) [14]. The PUFP measures UFP concentrations per cm^3^ of air every 1 s over concentrations of 0 – 2x10^5^ particles/cm^3^ (p/cc) with an accuracy of ± 10%. EcoMAPPE participants were instructed to wear the PUFP C200 for 3 h each day of sampling (the maximum battery life of the PUFP on a full charge). Additional details regarding the EcoMAPPE study and results have been described elsewhere [3,13].

We recruited adolescent focus group participants by stratifying the EcoMAPPE study population by self-reported race (White/non-White) and gender (male/female) and randomly contacting participants among each strata to ensure focus group participants were not the single representative of their race and gender. Recruitment was conducted by providing information about the study to the participant and caregivers by email with follow-up by phone. Caregivers of interested adolescents were also enrolled to form two focus groups of eight participants each. The size of each focus group was intentionally limited to eight to allow hands-on design and to encourage discussion. Ethical approval to conduct this research was obtained from the Cincinnati Children’s Hospital Institutional Review Board. Prior to participating in the focus groups, all caregivers and adolescents provided written informed consent and assent, respectively.

### Adolescent and Caregiver Focus Groups

We employed a qualitative approach to facilitate interactive discussion and co-design of report-back materials, with particular attention to content, design features, and communication strategies. Separate (adolescent and caregiver) in-person focus groups were semi-structured and designed to elicit meaningful qualitative feedback regarding their interest in receiving study results, perceived benefits and concerns, content, format, and delivery methods for reporting results back to participants and caregivers following personal air pollution monitoring. Adolescent and caregiver focus groups were scheduled to last one hour and facilitated by one qualitative researcher (AP and EH, respectively) and one member of the research study team. Focus groups were audio-recorded and transcribed verbatim.

Focus group guides were designed prior to meeting by the qualitative methodologists with additional input from the study team. Participant and caregiver focus group guides were similar, though the adolescent focus group guide included additional time for rapport building and activities to encourage participation. In both focus groups, participants were asked to describe the EcoMAPPE study to gain a better understanding of the words that they used to explain the research. Participants in the adolescent focus group were asked to design their own report-back materials, including drawing out an example of their own personal air pollution monitoring periods with relevant information to explain this to someone who had not participated in the study. Participants then did a “gallery walk” of the examples and wrote down the elements that they liked from each example. Participants also discussed how they would change their own map based on what others had drawn. In each of the focus groups, participants were provided examples of report-back documents that contained information about UFPs and prototype interactive graphs where users could toggle between different sampling days and asked to provide feedback on the materials including questions they had and suggestions for improvement.

### Expert Panel Review

We solicited feedback from a panel of four external experts in academia not involved with the EcoMAPPE study and with experience in community-engaged research, environmental health, personal exposure result reporting, and institutional review boards. Prior to a virtual meeting, the expert panel was provided with an electronic draft of the report-back materials developed following the initial focus groups of participants and caregivers. During a virtual semi-structured meeting, the expert panel reviewed all sections of the draft report-back material with the study team and provided suggestions for revisions that included changes to the language, figures, maps, colors, and information provided.

### Repeat Focus Group

**Fig. 1.**
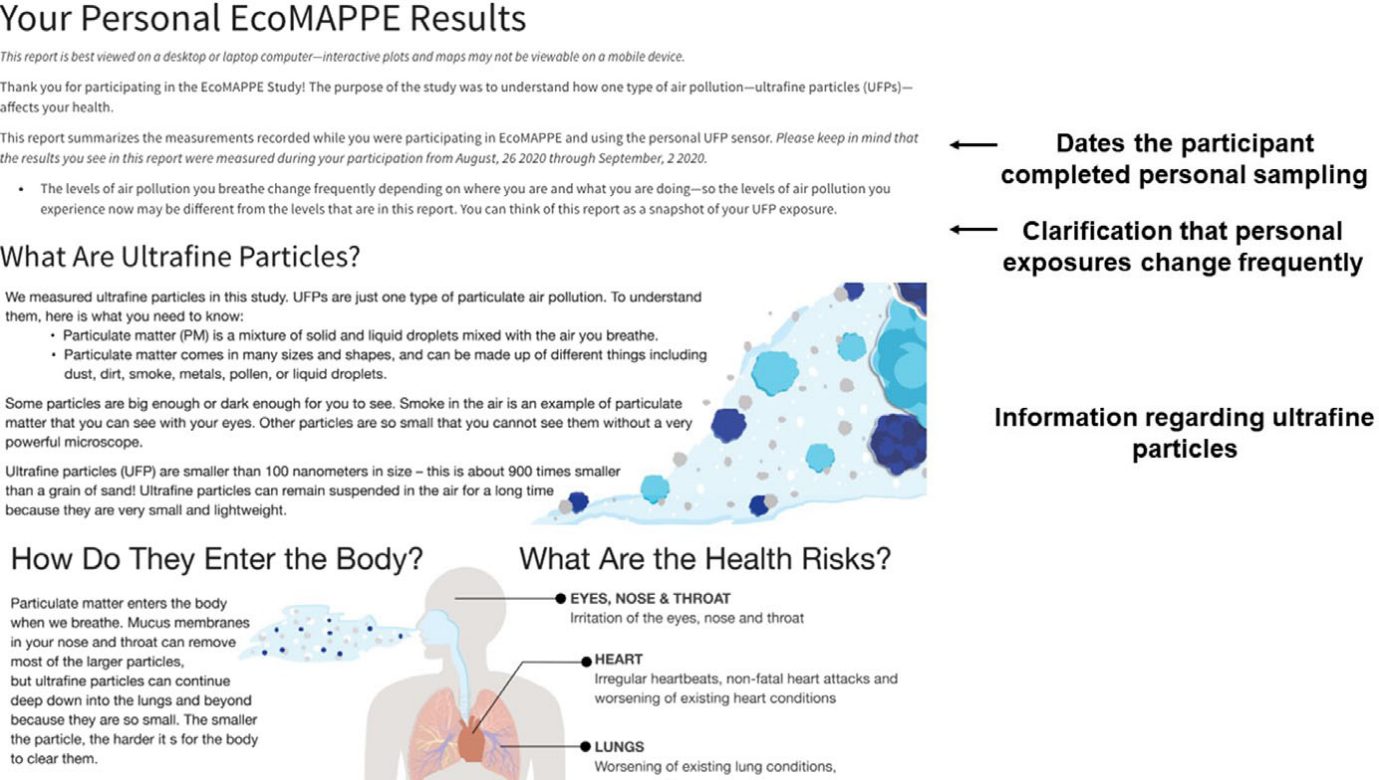
Introduction to the report-back document.

After revising the report-back document based on feedback from the expert panel, we reconvened the caregiver and adolescent focus groups in the summer of 2021 (Fig. 1). Due to the COVID-19 pandemic, the second focus groups were conducted virtually with separate sessions for adolescents and caregivers. In both focus groups, participants were provided a copy of the revised electronic report-back document followed by a guided discussion to elicit feedback on the presentation, content, readability, and usability of the report. Final revisions to the report-back document were made based on this feedback.

### Focus Group Analysis

We conducted a thematic analysis of the transcribed focus groups. Two members of the research study team and one data analyst external to the study made up the analysis team. All three analysts independently read the data line by line and recorded inductively derived open code (i.e., *in vivo* codes) for each relevant section of text in the data. The three data analysts reviewed codes together and came to consensus with the external team member providing feedback regarding assumptions and biases held by the research study team members. This process resulted in the development of a codebook that included examples of themes and subthemes. One research team member (AP) assigned the codes to themes and subthemes using inductive coding. The synthesized data were shared with the rest of the analysis team for feedback. Data and coding were organized using ATLAS.ti qualitative data analysis software.

### Report-Back Document Development

Based on focus group input, we designed the report-back document to be electronic and interactive. We developed a digital HTML report using R (4.1.1) and R Markdown (2.11) which incorporated instructional images and interactive plots and maps. Participant UFP sampling data were aggregated to 5-second median values for visualization, and the R packages “highcharter” (0.9.4) and “mapdeck” (0.3.4) were used to produce interactive plots and maps [15,16]. Standalone, instructional images were also included in the interactive report to provide background information on the personal sampler, UFPs, pollution sources, and reduction strategies.

## Results

### Focus Group Results

We enrolled a total of eight adolescents who participated in the EcoMAPPE study and one corresponding caregiver to provide their input on the report-back documents during focus group sessions. Adolescent participants were, on average, 14.4 years of age (range: 13–16 years) and one-half (n = 4) were female. Participants self-reported race included Black/Bi-racial (n = 2), White (n = 4), and more than one race (n = 2) and 25% (n = 2) reported having current asthma. Enrolled caregivers were, on average, 49 years of age, primarily female (n = 6), and White (n = 6). Five themes were derived from the coded focus group data and include: 1) feasibility and acceptability of the study, 2) visualizing data, 3) what participants want to know, 4) problems with the “black box,” and 5) utilization of the app (i.e., electronic report-back document).

When the focus group participants were asked to draw an example of how they would visualize their personal air pollution time periods, the prompt provided was “Think of an example of one of the times you wore the sensor. Using the paper in front of you sketch out a map of the time-period.” They were then asked to explain their map to the person next to them and adjust based on that conversation – what did they need to add or change to make the map understandable? The drawings from the adolescent focus groups revealed their preferences for the information included in report-back materials and how they would communicate that information. All participants drew the places that they went while in the study and many had some indication of travelling to and from each place. All participants indicated the level of pollution of each location using either color or density of dots or a combination of both. Two participants provided a key that indicated how the colors corresponded with the level of pollution and one of these participants provided a graph that indicated change in pollution as they traveled to and from different locations.

### Report-Back Document

Participants provided feedback on the visualization of the data on the mockup report-back materials. They shared which colors were better for visualizing the level of exposure to UFPs. One participant said, “when you’re in one spot and there are bigger [dots] and smaller [dots] that might be hard to see but with the color you can understand it a little better.” They also asked that that the report-back materials “map out the timeline” and the “direction we were traveling.” They also preferred illustrations to words, when possible and said things like, “it needs to be something that’s not childish but it still needs to be fun and catch attention.” Participants said they would “open it [the report-back document] up on my phone because I don’t actually have a computer with me and then go look at it on the computer because it’s easier.”

**Fig. 2.**
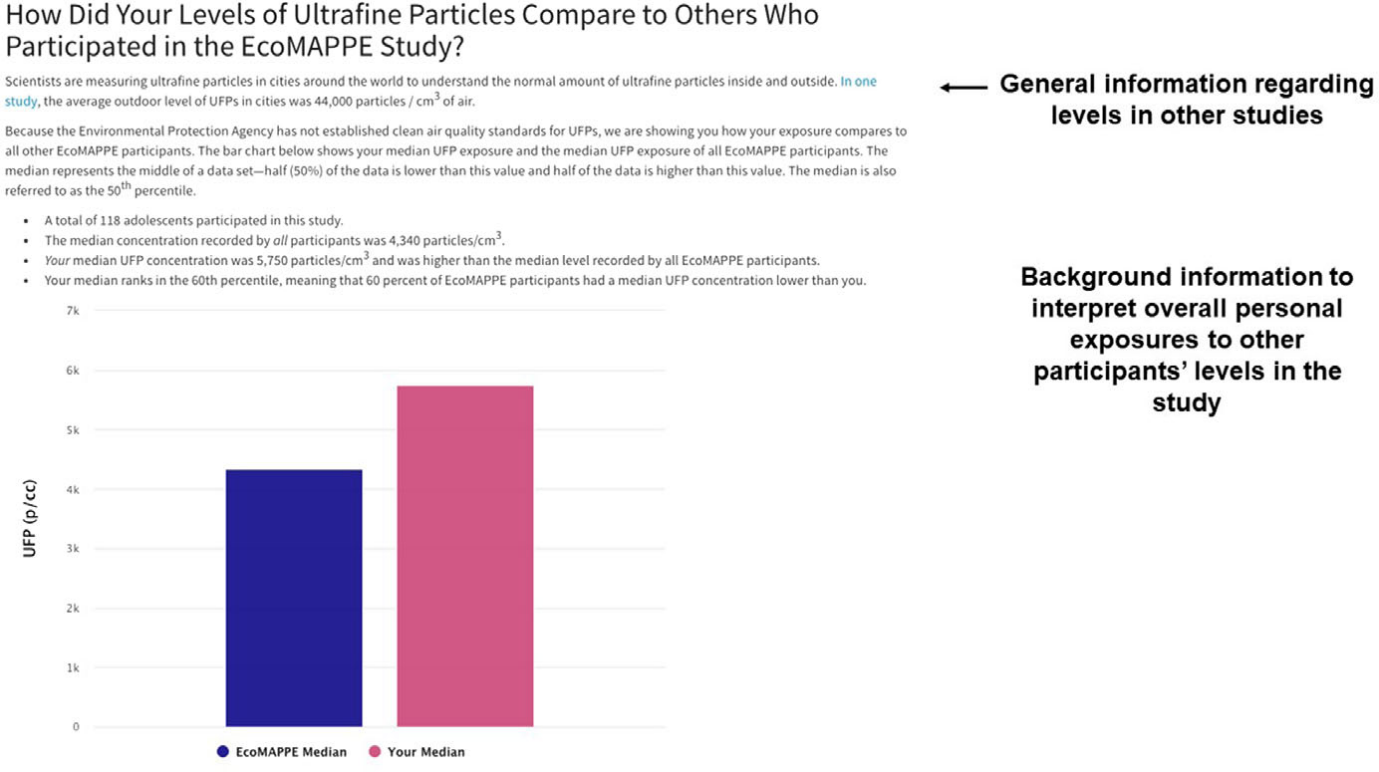
Comparison of participant’s median ultrafine particle exposure to the overall study median exposure.

**Fig. 3.**
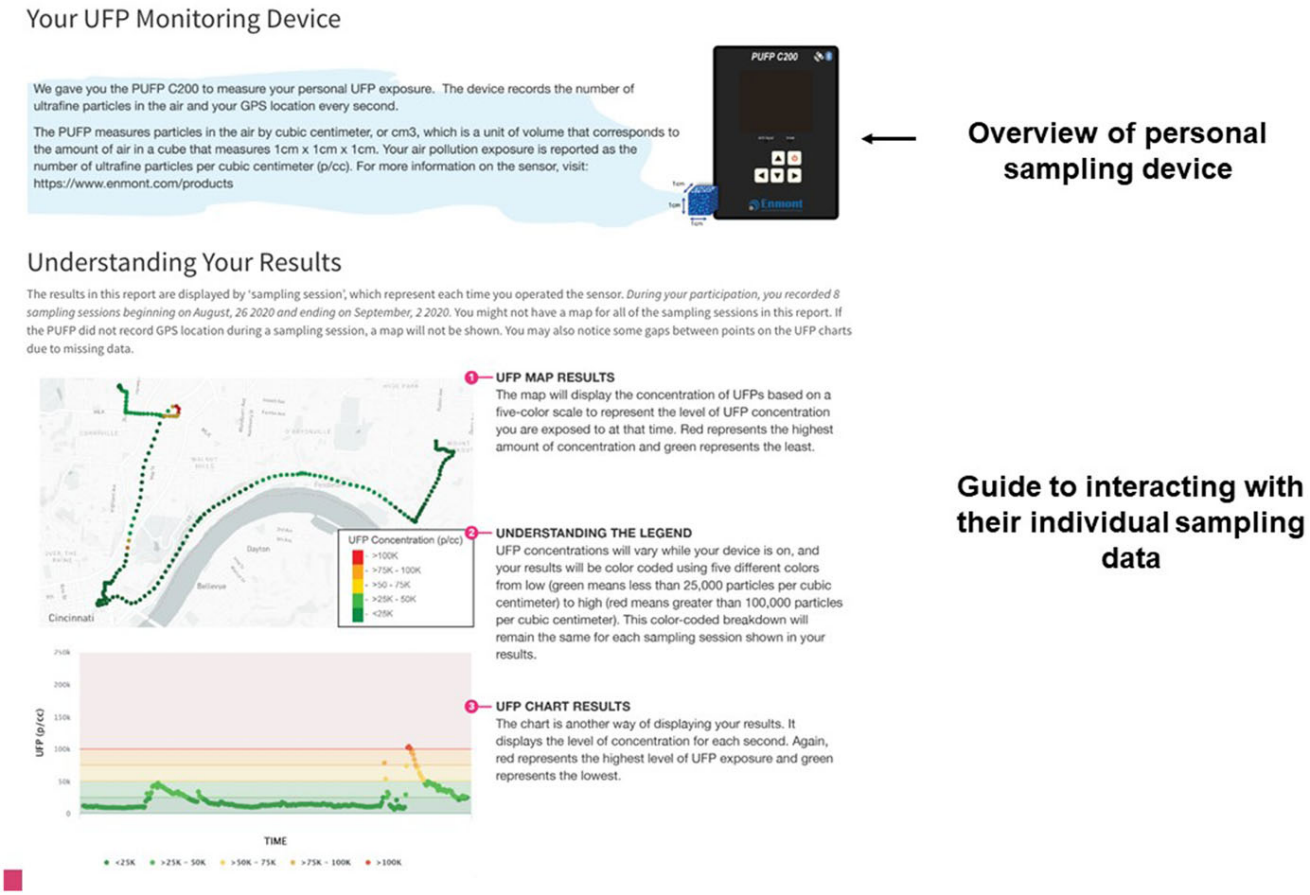
Guide to understanding the personal air sensor and results.

**Fig. 4.**
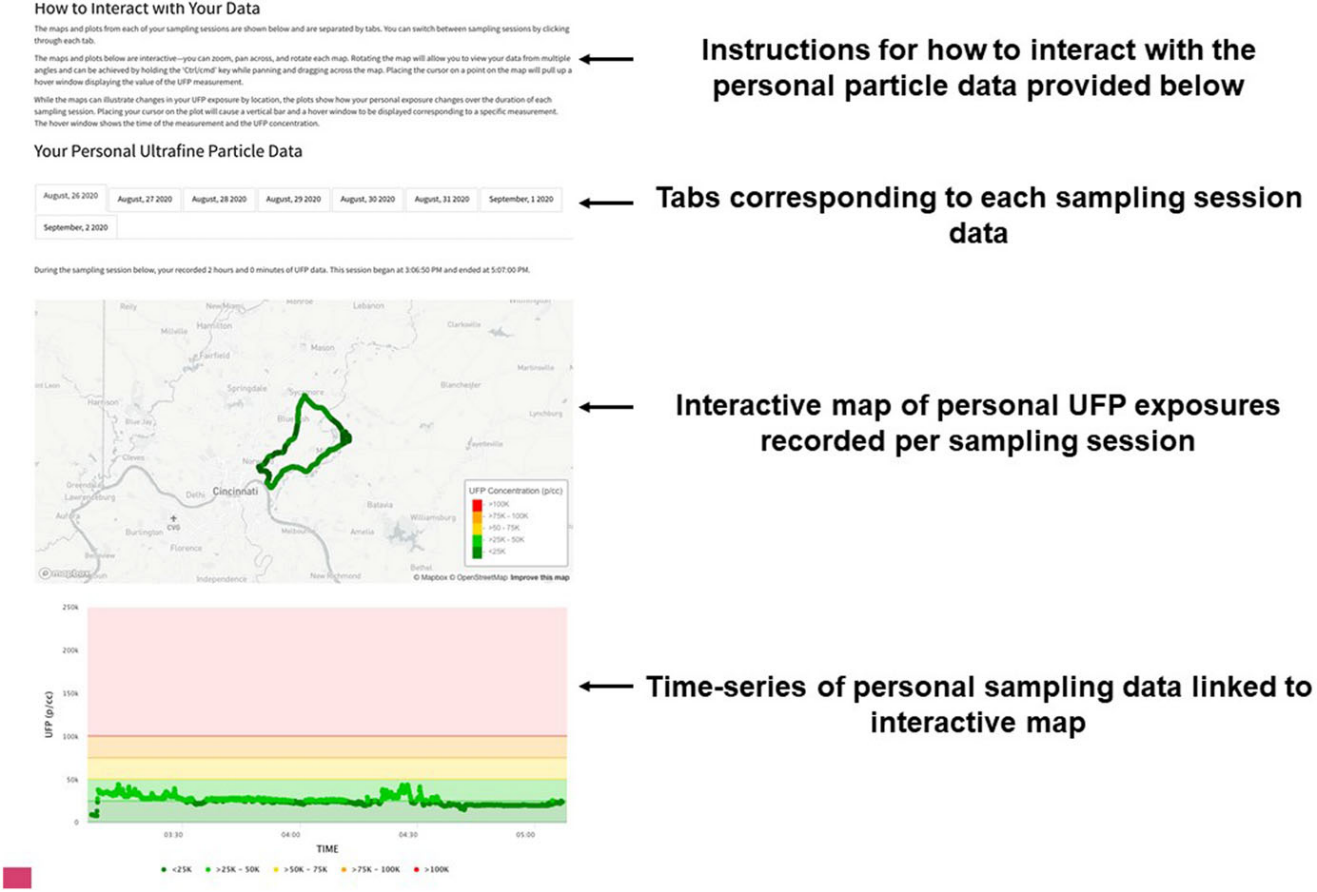
Interactive map and time series of personal air monitoring results for each day of personal sampling.

**Fig. 5.**
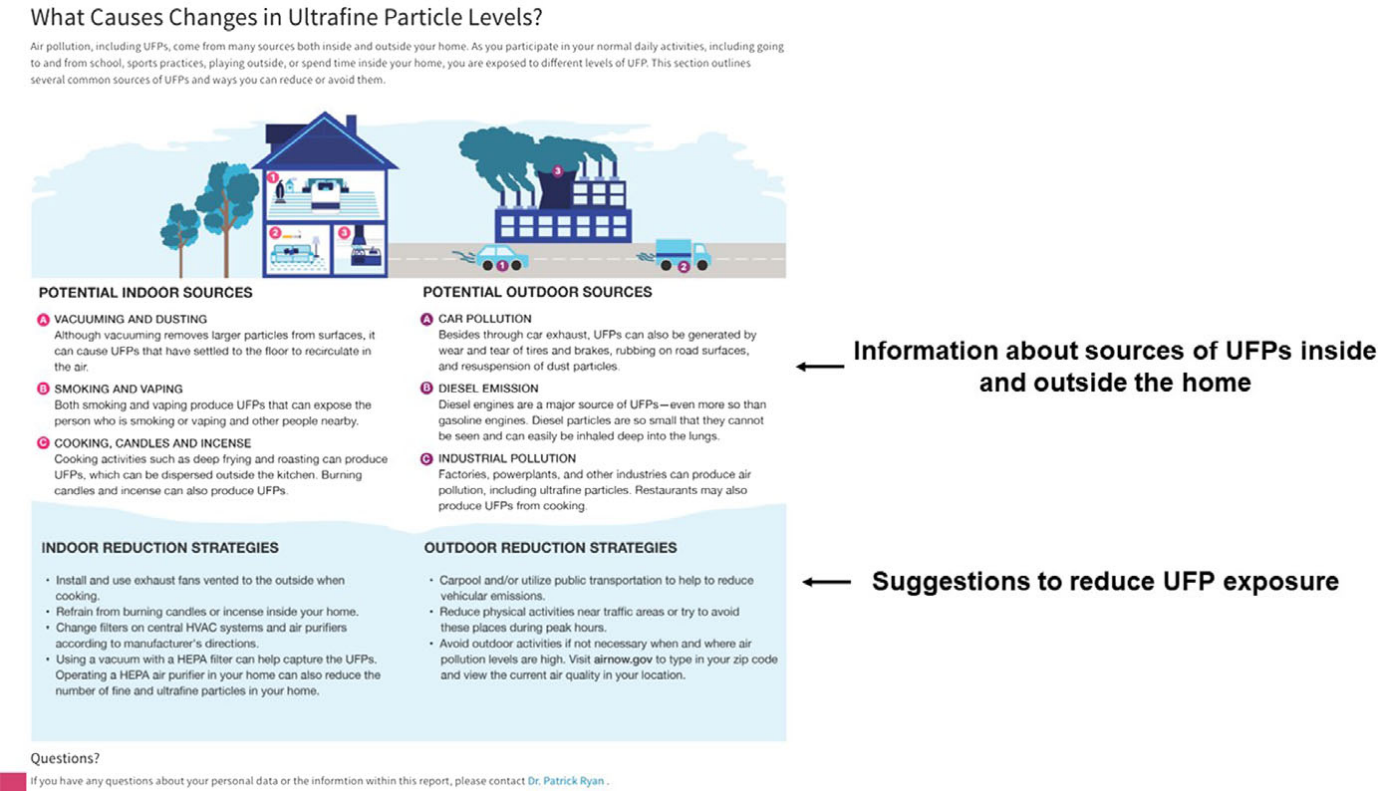
Additional information regarding sources of ultrafine particles and strategies to reduce personal exposure.

Based on this feedback from participants, caregivers, and experts, we produced a single web page to be provided electronically via email to the study participants and their caregivers. The HTML report-back document was composed of 1) an introduction to the materials including an overview of UFPs (Fig. 1), 2) a comparison of their median exposure compared to the entire study population (Fig. 2), 3) a guide to understanding the personal sensor and results (Fig. 3), 4) visualization of their personal sampling data using an interactive map and time-series of data corresponding to each day of personal sampling (Fig. 4), and 4) information on sources of UFPs, potential health risks, and strategies to reduce exposure (Fig. 5).

A key component to the report-back document included a statement that personal exposures change frequently based on many factors (Fig. 1). This point was emphasized by the expert panel as critical for the participants to recognize. Following a reminder of their dates of participation, the report-back document provides background information on UFPs, routes of exposure, and potential health risks. Language included in this section was informed by the focus group participants and the expert panel feedback from the expert panel. The first data provided to the participants are their overall median exposure levels recorded during the week of personal sampling (Fig. 2). Following discussion in the focus groups and expert panel, we determined a bar chart was the most informative approach to present this data. Prior to providing their overall median exposures, a brief definition of median is provided. Importantly, caregivers expressed a desire to place the overall levels of exposure in the context of other studies. Therefore, a general statement regarding outdoor exposure levels in other studies is also provided.

Prior to reporting the results of individual real-time sampling data, a summary of the PUFP sensor is provided (Fig. 3). Then, an informative guide to understand the maps and time-series data is presented (Fig. 3) so that participants can interpret their personal sampling data. This includes a color scale, which represent UFP exposure categories ordered from low (green; < 25K p/cc) to high (red; > 100K p/cc). As shown in Fig. 4, instructions for how to interact with their personal particle data is provided. Participants can explore the map of their personal exposures by zooming in and out and panning across the locations of each measurement. Participants can also rotate the map to visualize the locations in 3D. Maps and corresponding time-series plots also display additional information when the user places their cursor or taps on a data point. For the maps, the UFP concentration is displayed. Likewise, the UFP concentration, date and time of the measurement, and a vertical bar highlighting the data point are displayed while hovering on the time-series plot. Separate tabs corresponding to each sampling period are available so that participants can visualize the results of their sampling by day of participation. The report-back document concludes with additional information regarding indoor and outdoor sources of UFPs and potential strategies to reduce exposures (Fig. 5).

## Discussion

To our knowledge, previous studies of personal air pollution exposure have not examined participants’ desire to receive their results nor considered best practices to return these results using an understandable and actionable approach. In this study, we engaged adolescents who completed personal air sampling for UFPs, their caregivers, and academic experts to co-design report-back materials that is appropriate and specific to the study population – a critical component to developing report-back documents [5,12]. Using this approach, we identified key components to the report-back document including background information about UFPs and the device that was used to measure their exposure, providing a summary of individual results compared to others, an interactive visualization of their personal monitoring results, and information on sources of exposure, reduction strategies, and health risks.

Importantly, our report-back document was created as an HTML document based on our participants desire to receive their data in electronic format. In addition to being the preferred method of adolescents to receive their study results, an electronic document provides some additional benefits compared to a written or printed report. For example, we were able to embed interactive maps and figures into the electronic HTML document which allows the viewer to zoom in to specific locations and times of interest. In addition, electronic documents may enhance readability for adolescents as information can be tabulated of separate pages or links to decrease the overall length of the document. Finally, electronic documents may be generated and distributed in shorter time frames allowing for results to be provided with smaller delays from the time of data collection to dissemination.

In response to participants in biomonitoring studies desire to receive their results, guidelines for providing biomonitoring results back to study participants are now available [9,10]. There are, however, important differences between biomonitoring studies and data collected by personal air monitors at a high resolution over time that necessitated this study. For example, biomonitoring studies typically collect a limited number of biospecimens (e.g., blood, urine) to assess the presence of chemicals with varying half-lives. Frequently, months or years may pass from the time of sample collection to return of biomonitoring results due to the time required to perform laboratory assays. In contrast, personal air monitors measure individuals’ exposure with high temporal resolution (e.g., every 1 s) and can be combined with Bluetooth or other technology to facilitate immediate data transfer to the study participant or researcher. This temporal resolution, when combined with GPS-captured location, provides a unique opportunity to identify places, activities, and times with elevated personal exposures. Thus, personal air monitoring provides the user actionable information to raise awareness and guide behavioral changes to reduce exposure [17,18]. These behavioral changes offer the potential to significantly reduce overall exposure to air pollution given that short term but high air pollution events contribute a disproportionate percentage of individuals’ total daily exposure [14,19–24]. Furthermore, behavioral modifications to reduce exposures, including modifying cycling and pedestrian routes, can significantly reduce personal UFP, black carbon, and particulate matter (PM) exposures [25,26].

Providing study participants their individual results may also lead to increased trust in science, environmental health literacy, individual empowerment, and motivation to reduce environmental exposures [9]. Engaging participants by providing them the results of their research provides opportunity to progress from recognizing the relationship between exposure and health, to actively reducing exposure and improving health [12]. Reporting results to participants also facilitates a collaborative research approach where participants not only provide data but also receive feedback. This engagement can enhance recruitment and retention, detect novel sources of exposure, and identify new research topics [6,27].

In addition to engaging with participants through focus groups, our study had several additional strengths including the input of experts experienced in community-engaged research, human subjects’ research, and personal air sampling but not associated with the EcoMAPPE study. These researchers provided external perspective and suggestions on our report-back materials prior to our second focus groups. Our focus on UFPs, an air pollutant hypothesized to be more toxic than larger particles but whose human health effects are understudied [28–32], has also allowed us to develop reports of exposure to a pollutant with unknown health effects. Finally, we provided the participants with interactive maps and corresponding time-series data enabling them to identify specific times and locations where their personal exposures were elevated or decreased. Future research will examine whether providing participants their individual results increase their knowledge and awareness of environmental health, air pollution, and motivates changes in behavior to reduce air pollution exposures.

## References

[1] Johnston JE , Juarez Z , Navarro S , Hernandez A , Gutschow W. Youth engaged participatory air monitoring: a ‘Day in the Life’ in urban environmental justice communities. International Journal of Environmental Research and Public Health 2019; 17(1): 93. 31877745 10.3390/ijerph17010093 PMC6981490

[2] Nieuwenhuijsen MJ , Donaire-Gonzalez D , Foraster M , Martinez D , Cisneros A. Using personal sensors to assess the exposome and acute health effects. International Journal of Environmental Research and Public Health 2014; 11: 7805–7819. 25101766 10.3390/ijerph110807805 PMC4143834

[3] Turner AL , Brokamp C , Wolfe C , Reponen T , Ryan PH. Impact of personal, sub-hourly exposure to ultrafine particles on respiratory health in adolescents with asthma. Annals of the American Thoracic Society 2022; 19(9): 1516–1524. DOI: 10.1513/AnnalsATS.202108-947OC. 35315743 PMC9447389

[4] Larkin A , Hystad P. Towards personal exposures: how technology is changing air pollution and health research. Current Environmental Health Reports 2017; 4(4): 463–471. DOI: 10.1007/s40572-017-0163-y. PMC5677549 28983874

[5] Ohayon JL , Cousins E , Brown P , Morello-Frosch R , Brody JG. Researcher and institutional review board perspectives on the benefits and challenges of reporting back biomonitoring and environmental exposure results. Environmental Research 2017; 153: 140–149. 27960129 10.1016/j.envres.2016.12.003 PMC5412511

[6] Ramirez-Andreotta MD , Brody JG , Lothrop N , Loh M , Beamer PI , Brown P. Improving environmental health literacy and justice through environmental exposure results communication. International Journal of Environmental Research and Public Health 2016; 13(7): 690. 27399755 10.3390/ijerph13070690 PMC4962231

[7] Adams C , Brown P , Morello-Frosch R , et al. Disentangling the exposure experience: the roles of community context and report-back of environmental exposure data. J Health Soc Behav 2011; 52: 180–196. 21673146 10.1177/0022146510395593 PMC3175404

[8] Wu N , McClean MD , Brown P , Aschengrau A , Webster TF. Participant experiences in a breastmilk biomonitoring study: a qualitative assessment. Environmental Health 2009; 8: 4. 19226469 10.1186/1476-069X-8-4 PMC2649062

[9] Brody JG , Dunagan SC , Morello-Frosch R , Brown P , Patton S , Rudel RA. Reporting individual results for biomonitoring and environmental exposures: lessons learned from environmental communication case studies. Environmental Health 2014; 13: 40. 24886515 10.1186/1476-069X-13-40 PMC4098947

[10] Dunagan S , Brody J , Morello-Frosch R , et al. When Pollution is Personal: Handbook for Reporting Results to Participants in Biomonitoring and Personal Exposure Studies. Newton, MA: Silent Spring Institute, 2013.

[11] Brody JG , Cirillo PM , Boronow KE , et al. Outcomes from returning individual versus only study-wide biomonitoring results in an environmental exposure study using the Digital Exposure Report-Back Interface (DERBI). Environmental Health Perspectives 2021; 129: 117005. 34766835 10.1289/EHP9072 PMC8589017

[12] Finn S , O’Fallon L. The emergence of environmental health literacy—from its roots to its future potential. Environmental Health Perspectives 2017; 125: 495. 26126293 10.1289/ehp.1409337 PMC5382009

[13] Turner A , Brokamp C , Wolfe C , Reponen T , Ryan P. Personal exposure to average weekly ultrafine particles, lung function, and respiratory symptoms in asthmatic and non-asthmatic adolescents. Environment International 2021; 156: 106740. 34237487 10.1016/j.envint.2021.106740 PMC8380734

[14] Ryan PH , Son SY , Wolfe C , Lockey J , Brokamp C , LeMasters G. A field application of a personal sensor for ultrafine particle exposure in children. Science of the Total Environment 2015; 508: 366–373. 25497676 10.1016/j.scitotenv.2014.11.061

[15] Kunst J. (2022) *Highcharter: a wrapper for the ‘Highcharts’ library. R package version 094*. (https://CRAN.R-project.org/package=highcharter)

[16] Cooley D. (2020) *mapdeck: Interactive Maps Using ‘Mapbox GL JS’ and ‘Deck.gl’. R package version 0.3.4*. (https://CRAN.R-project.org/package=mapdeck)

[17] Steinle S , Reis S , Sabel CE. Quantifying human exposure to air pollution—moving from static monitoring to spatio-temporally resolved personal exposure assessment. Science of the Total Environment 2013; 443: 184–193. 23183229 10.1016/j.scitotenv.2012.10.098

[18] Steinle S , Reis S , Sabel CE , et al. Personal exposure monitoring of PM 2.5 in indoor and outdoor microenvironments. Science of the Total Environment 2015; 508: 383–394. 25497678 10.1016/j.scitotenv.2014.12.003

[19] Van Ryswyk K , Wheeler AJ , Wallace L , et al. Impact of microenvironments and personal activities on personal PM2. 5 exposures among asthmatic children. J Expo Sci Environ Epidemiol 2014; 24: 260–268. 23632991 10.1038/jes.2013.20

[20] Zuurbier M , Hoek G , Oldenwening M , Meliefste K , van den Hazel P , Brunekreef B. Respiratory effects of commuters’ exposure to air pollution in traffic. Epidemiology 2011; 22: 219–227. 21228698 10.1097/EDE.0b013e3182093693

[21] Buonanno G , Stabile L , Morawska L. Personal exposure to ultrafine particles: the influence of time-activity patterns. Sci Total Environ 2014; 468–469: 903–907. 10.1016/j.scitotenv.2013.09.016 24080417

[22] Diapouli E , Chaloulakou A , Spyrellis N. Levels of ultrafine particles in different microenvironments--implications to children exposure. Science of the Total Environment 2007; 388: 128–136. 17888492 10.1016/j.scitotenv.2007.07.063

[23] Dons E , Int Panis L , Van Poppel M , Theunis J , Wets G. Personal exposure to black carbon in transport microenvironments. Atmospheric Environment 2012; 55: 392–398.

[24] Buonanno G , Stabile L , Morawska L , Russi A. Children exposure assessment to ultrafine particles and black carbon: The role of transport and cooking activities. Atmospheric Environment 2013; 79: 53–58.

[25] Cole-Hunter T , Jayaratne R , Stewart I , Hadaway M , Morawska L , Solomon C. Utility of an alternative bicycle commute route of lower proximity to motorised traffic in decreasing exposure to ultra-fine particles, respiratory symptoms and airway inflammation–a structured exposure experiment. Environmental Health 2013; 12: 29. 23566176 10.1186/1476-069X-12-29 PMC4177132

[26] Hankey S , Lindsey G , Marshall JD. Population-level exposure to particulate air pollution during active travel: planning for low-exposure, health-promoting cities. Environmental Health Perspectives 2017; 125: 527. 27713109 10.1289/EHP442 PMC5381994

[27] Ramirez-Andreotta MD , Brody JG , Lothrop N , Loh M , Beamer PI , Brown P. Reporting back environmental exposure data and free choice learning. Environmental Health 2016; 15: 2. 26748908 10.1186/s12940-015-0080-1 PMC4707004

[28] Elder A , Oberdörster G. Translocation and effects of ultrafine particles outside of the lung. Clin Occup Environ Med 2005; 5: 785–796. 10.1016/j.coem.2006.07.003 17110292

[29] Kumar S , Verma MK , Srivastava AK. Ultrafine particles in urban ambient air and their health perspectives. Rev Environ Health 2013; 28: 117–128. 24192498 10.1515/reveh-2013-0008

[30] Oberdörster G , Oberdörster E , Oberdörster J. Nanotoxicology: an emerging discipline evolving from studies of ultrafine particles. Environmental Health Perspectives 2005; 113: 823. 16002369 10.1289/ehp.7339 PMC1257642

[31] Oberdorster G , Sharp Z , Atudorei V , et al. Translocation of inhaled ultrafine particles to the brain. Inhal Toxicol 2004; 16: 437–445. 15204759 10.1080/08958370490439597

[32] HEI Review Panel on Ultrafine Particles. Understanding the Health Effects of Ambient Ultrafine Particles. HEI Perspectives 3. Boston, MA: Health Effects Institute, 2013.

